# Blueberry (*Vaccinium* spp.) Anthocyanins and Their Functions, Stability, Bioavailability, and Applications

**DOI:** 10.3390/foods13172851

**Published:** 2024-09-08

**Authors:** Li Wang, Wei Lan, Dan Chen

**Affiliations:** 1 Anhui Ecological Fermentation Engineering Research Center for Functional Fruit Beverage, Fuyang Normal University, Fuyang 236037, China; lwang@ahau.edu.cn (L.W.); lanwei@fynu.edu.cn (W.L.); 2 College of Tea and Food Science and Technology, Anhui Agricultural University, Hefei 210036, China; 3 College of Food Science and Engineering, Yangzhou University, Yangzhou 225127, China

**Keywords:** blueberry, anthocyanins, beneficial effects, bioaccessibility

## Abstract

Blueberry fruits are rich in anthocyanins. There are 25 known anthocyanidins found in blueberries (*Vaccinium* spp.) until now. Anthocyanins found in blueberries have attracted considerable interest for their outstanding abilities as antioxidants, anti-inflammatory agents, anti-diabetic, anti-obesity, and neuroprotection compounds, as well as their potential for preventing cardiovascular diseases, protecting vision, and inhibiting cancer development. However, their application is constrained by issues related to instability and relatively low bioavailability. Thus, this review provides a detailed overview of categories, functions, stability, and bioavailability of blueberry anthocyanins and their practical applications. The available studies indicate that there is more potential for the industrial production of blueberry anthocyanins.

## 1. Introduction

Blueberries are part of the *Vaccinium* genus within the Ericaceae family, which comprises around 450 species worldwide [1]. Blueberries, a perennial shrub originally from North America, are now grown in different areas including China, Japan, Chile, Europe, Argentina, New Zealand, and Australia [2]. The cultivated blueberry varieties can be categorized into three main types based on plant size and cold storage requirements: highbush blueberries (*Vaccinium corymbosum* L.), lowbush blueberries (*V. angustifolium* Ait.), and rabbit-eye blueberries (*V. ashei* Reade) [3]. Blueberry fruits are globose, dark blue, glabrous, and whitish [4]. Blueberry fruits are abundant in essential micronutrients, prebiotic fibers, vitamins, and bioactive polyphenols, while also containing few calories and having a high level of hydration [5]. They also contain high levels of anthocyanins and are popular worldwide for their delicious flavor [3].

**Figure 1:**
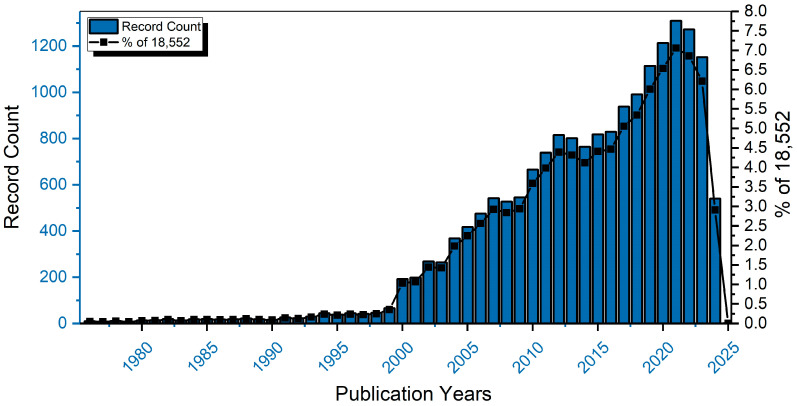
The yearly count of online publications was obtained from the Web of Science (WOS, Thomson Reuters Company) database spanning from 1976 to 2024 (Topic = “Vaccinium” or “blueberry” and “Anthocyanin”).

Blueberry anthocyanins have long been of interest to researchers. The quantity of papers focusing on blueberry anthocyanins has experienced a significant surge in the last two decades (Figure 1). A search on the Web of Science (WOS, Thomson Reuters Company) database for topics including “*Vaccinium*” or “blueberry” and “Anthocyanin” yielded a total of 18,552 results (until July 2024). There is a strong interest in blueberry anthocyanins because of their possible therapeutic and beneficial effects, including lowering of coronary artery disease, anti-cancer/anti-tumor, anti-inflammatory, anti-diabetic, and improving vision and cognitive behavior. The present study is focused on analyzing the categories, structure, and content of blueberry anthocyanins; summarizing the important roles and health advantages of these substances; and investigating the current application and potential future prospects of anthocyanins form blueberries. This research aims to provide valuable insights for the improved utilization of blueberry resources.

## 2. Anthocyanins in *Vaccinium* spp.

Anthocyanins, a type of flavonoid, are composed of two aromatic rings, A and B, linked by a chain consisting of a C-ring with three carbon atoms. Structural variations in the B-ring determine the class of anthocyanins [3]. Glycosides typically affiliate with the 3-position on the C-ring (resulting in 3-monoglycosides) or the 5-position on the A-ring (resulting in 3,5-diglycosides) [6]. Different glycosidic units (mono-, di-, and tri-glycosides are present in anthocyanin structures [7]. The predominant monoglycosides are hexoses (galactose, glucose, or rhamnose), or pentoses (arabinose and xylose). There are 25 known anthocyanidins found in blueberries until now, including malvidin-3-*O*-galactoside, malvidin-3-*O*-arabinoside, cyanidin-3-*O*-galactoside, cyanidin-3-*O*-arabinoside, paeonidin-3-*O*-galactoside, petunidin 3-*O*-glucoside, petunidin-3-*O*-galactoside, malvidin-3-*O*-glucoside, delphinidin-3-*O*-galactoside, petunidin-3-*O*-arabinoside, delphinidin-3-*O*-arabinoside, cyanidin-3-*O*-glucoside, delphinidin-3-*O*-glucoside, and eleven acylated structures (Table 1) [7,8]. 

Blueberries contain various types of anthocyanins, but their impact on the overall phenolic concentration and the variety of anthocyanin categories can differ due to elements such as the variety of the plant, the size of the berries, and environmental factors like sunlight and irrigation [9,10]. From the anthocyanin content of fresh blueberry fruits, malvidin glycosides are most abundant compared to other types of anthocyanins, followed by cyanidin, petunidin, paeonidin, and delphinidin glycosides. Nevertheless, the preservation rate of anthocyanin in dried fruits differs significantly based on the method of drying, with freeze-drying showing the highest preservation rate [8]. Nonetheless, blueberry anthocyanins are dominated by malvidin-3-*O*-galactoside and malvidin-3-*O*-arabinoside.

**Table 1:** Anthocyanins from blueberry fruit [3,7,11].

Chemical Structure	Anthocyanin Profiles	R_1_	R_2_	R_3_	Content (mg/100 g)	
					Fresh Fruits	Dried Fruits
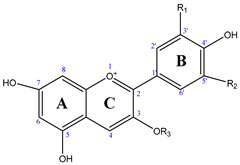	cyanidin-3-*O*-arabinoside	OH	H	Ara	14.33	1.08–52.8
	cyanidin-3-*O*-galactoside	OH	H	Gal	19.1–28.1	2.76–49.3
	cyanidin-3-*O*-glucoside	OH	H	Glc	0.62–11.7	0.12–10.5
	cyanidin-3-*O*-(6″-acetyl)glucoside	OH	H	^6″Ac^Glc	-	0–7.5
	cyanidin-3-*O*-(6″-acetyl)arabinoside	OH	H	^6″Ac^Ara	-	-
	cyanidin-3-*O*-(6″-acetyl)galactoside	OH	H	^6″Ac^Gal	-	-
	delphinidin-3-*O*-arabinoside	OH	OH	Ara	3.73–13.3	0.48–39.2
	delphinidin-3-*O*-galactoside	OH	OH	Gal	5.61–26.9	1.08–81.4
	delphinidin-3-*O*-glucoside	OH	OH	Glc	0–10.1	0–38.7
	delphinidin-3-*O*-(6″-acetyl)glucoside	OH	OH	^6″Ac^Glc	-	0–25.1
	delphinidin-3-*O*-(6″-acetyl)galactoside	OH	OH	^6″Ac^Gal	-	-
	malvidin-3-*O*-arabinoside	OCH_3_	OCH_3_	Ara	13.3–42.38	2.52–54.3
	malvidin-3-*O*-galactoside	OCH_3_	OCH_3_	Gal	23.4–66.68	5.29–108.8
	malvidin-3-*O*-glucoside	OCH_3_	OCH_3_	Glc	8.72–37.2	0.60–86.1
	malvidin-3-*O*-(6″-acetyl)galactoside	OCH_3_	OCH_3_	^6″Ac^Gal	-	0.1–9.1
	malvidin-3-*O*-(6″-acetyl)glucoside	OCH_3_	OCH_3_	^6″Ac^Glc	-	0.1–21.4
	paeonidin-3-*O*-galactoside	OCH_3_	H	Gal	12.46–20.9	1.32–19.8
	paeonidin-3-*O*-glucoside	OCH_3_	H	Glc	11.9–12.0	-
	paeonidin-3-*O*-(6″-acetyl)galactoside	OCH_3_	H	^6″Ac^Gal	-	-
	paeonidin-3-*O*-(6″-acetyl)glucoside	OCH_3_	H	^6″Ac^Glc	-	-
	petunidin-3-*O*-arabinoside	OCH_3_	OH	Ara	5.61–9.05	0.48–21.3
	petunidin 3-*O*-glucoside	OCH_3_	OH	Glc	3.5–28.1	0.5–37.9
	petunidin-3-*O*-galactoside	OCH_3_	OH	Gal	8.72	1.2–34.0
	petunidin-3-*O*-(6″-acetyl)galactoside	OCH_3_	OH	^6″Ac^Gal	-	0–10.2
	petunidin-3-*O*-(6″-acetyl)glucoside	OCH_3_	OH	^6″Ac^Glc	-	-

- No data available. Ac, acetyl; Ara, arabinoside; Gal, galactoside; Glc, glucoside.

## 3. Functions of Blueberry Anthocyanins

Blueberry anthocyanins have sparked significant research interest and received widespread recognition in the scientific field because of their remarkable properties that promote good health. An increasing amount of studies suggest that blueberry anthocyanins have been shown to demonstrate various pharmacological effects, including antioxidative properties, reduction in glucose levels, anti-cancer activity, inflammation and cardiovascular disease risk reduction, and neuroprotective effects. The wide variety of health benefits suggests that blueberry anthocyanins have potential for use in various fields.

### 3.1. Antioxidant

The predominant factors in blueberries that contribute to the antioxidant capacity of active compounds are generally acknowledged to be anthocyanidins, accounting for around 84% of the total antioxidant capacity [12]. The powerful antioxidant properties of blueberry anthocyanins have been extensively studied and confirmed in both laboratory experiments and living organisms (Table 2). Anthocyanins helps neutralize free radicals and reduce oxidative stress by giving up hydrogen atoms from the phenolic ring [13,14]. Zhou et al. [15] reported that high-purity blueberry anthocyanins (containing 14 anthocyanins) showed a median effective concentration (EC_50_) value of 14.99 μg/mL for scavenging ABTS^•+^ radicals and 26.48 μg/mL for scavenging DPPH radicals. Li et al. [16] conducted a study to assess the antioxidant capacity of anthocyanins in vitro from ten different varieties of blueberries (*Blomidon*, *Cumberland*, *Chippewa*, *Fundy*, *Imer*, *Northblue*, *Northlan*, *Polaris*, *Patriot*, *Rekad*). Among them, Polaris’s anthocyanins had the largest amounts of anthocyanins, thus showing the highest antioxidant abilities against acrylamide-induced toxicity in HepG2 cell. Experiments on animals and cells have demonstrated that blueberry anthocyanins possess antioxidant properties. This has been evidenced by their capacity to reduce lipid peroxidation in mice with a vitamin E deficiency (ApoE^-/-^) and enhance protective effects in cultured endothelial cells [17,18,19]. It was discovered that the extract of blueberry anthocyanin, specifically malvidin, malvidin-3-glucoside, and malvidin-3-galactoside, can reduce oxidative stress in human retinal pigment epithelial (RPE) cells by decreasing the levels of malondialdehyde (MDA) and reactive oxygen species (ROS) while increasing levels of catalase (CAT), superoxide dismutase (SOD), and glutathione peroxidase (GPx). The bioactivities were found to involve mitogen-activated-protein-kinase (MAPK) pathways, including ERK1/2 and p38 [18]. An additional study found that the primary anthocyanins in blueberries (malvidin-3-galactoside and malvidin-3-glucoside) were associated with a reduction in levels of ROS and xanthine oxidase-1 (XO-1), while increasing the levels of SOD and heme oxygenase-1 (HO-1) in endothelial cells [20]. Interestingly, glycosides significantly enhanced the antioxidant potential of malvid. Some research suggests that the biological effects of blueberry anthocyanins may not be solely attributed to their chemical properties. Multiple investigations have indicated that the anthocyanins present in blueberries possess antioxidant properties, resulting from intricate biochemical processes. These processes involve the upregulation of antioxidant enzyme activities or the downregulation of oxidase expression. In research conducted by Wu et al. [17], it was found that mice showed elevated levels of antioxidant enzymes like SOD1, SOD2, thioredoxin reductase 1 (TXNRD1), and glutathione reductase (GSR) after consuming freeze-dried blueberry powder for 20 weeks. Song et al. [19] discovered that at a concentration of 50 μM, delphinidin is capable of decreasing the expression of different subunits of nicotinamide adenine dinucleotide phosphate (NADPH) oxidase, which plays a role in controlling the generation of ROS. Research has indicated that the anthocyanins found in blueberries have the ability to safeguard cells from harm caused by ROS, which are associated with long-term illnesses like cardiovascular diseases (CVD) and diabetes [21,22]. Besides, blueberry anthocyanin extract was found to suppress the accumulation of ROS induced by acrylamide and the depletion of glutathione (GSH), enhance the movement of sperm, and reduce the percentage of abnormal sperm [23]. The activities of GPx and glutathione-S-transferase (GST) were also enhanced, along with increased protein expression of GPx, GST, and gamma-glutamyl cysteine synthase (γ-GCS), while the protein expression of cytochrome P450 2E1 (CYP2E1) was inhibited.

### 3.2. Anti-Inflammatory

Chronic inflammation is strongly associated with CVD, diabetes, arthritis, and other chronic illnesses and plays a major role in the onset of these conditions [14]. Multiple research studies have indicated that both blueberry fruits and their anthocyanins are capable of modulating the inflammatory process (Table 2). As a result, blueberry anthocyanins can alleviate and/or prevent related chronic diseases. During cellular tests, the anthocyanin extract from blueberries successfully suppressed the inflammatory response of Raw 264.7 macrophages induced by lipopolysaccharide (LPS) by inhibiting the mRNA levels of inflammatory biomarkers like IL-1β, COX-2, and iNOS [24]. During animal experiments, the use of blueberry extract containing 0.58 mg/mL of anthocyanin resulted in a decrease in the development of acute inflammation and arthritis clinical symptoms (such as bone absorption, osteophyte formation, and soft tissue swelling) in collagen-induced arthritis rats [25]. Oral administration of blueberry anthocyanins at a dosage of 80 mg/kg in STZ-induced diabetic rats resulted in a significant reduction in the serum levels of interleukin-1β (IL-1β) and vascular endothelial growth factor (VEGF) [22]. The mice treated with a complex of high hydrostatic pressure cyanidin-3-glucoside and blueberry pectin showed an effective anti-inflammatory response with dextran sodium sulfate-induced ulcerative colitis [26]. A study involving 2375 participants discovered that a higher consumption of anthocyanins, such as those found in blueberries, was linked to a reduction in the levels of twelve various markers related to inflammation [27]. In Raw 264.7 macrophages, blueberry polyphenol extract was found to inhibit the activation of the NF-κB pathway and MAPK pathway, leading to a decrease in TNF-α and IL-6 [28].

### 3.3. Anti-Diabetic

Blueberry anthocyanins have shown promise in managing diabetes and metabolic syndrome. They can improve insulin sensitivity, modulate glucose metabolism, and reduce the risk of complications associated with diabetes, such as neuropathy and retinopathy. Palma et al. [29] discovered that the consumption of fresh blueberries may enhance the management of post-meal glucose levels, potentially as a result of their impact on the digestive system. Additionally, blueberries supplementation can enhance insulin sensitivity, probably due to its properties of antioxidant and anti-inflammatory. Insulin resistance and comparatively reduced insulin production are contributing factors to the development of type 2 diabetes, which in turn results in increased blood glucose levels. A diet containing 8% enriched blueberry powder was given to obese Zucker rats, resulting in significant reductions in the levels of retinol binding protein 4 (RBP4) plasma glycosylated hemoglobin HbA1c and resistin [30]. In C57B1/6J mice with diabetes, both a blueberry extract containing high levels of anthocyanin (595 mg/g) and purified malvidin were found to effectively reduce hyperglycemia [31]. In men with type 2 diabetes, the consumption of 22 g freeze-dried blueberry for a period of 8 weeks may have positive effects on cardiometabolic health parameters [32]. The potential treatment of type 2 diabetes with blueberry anthocyanins may involve inhibiting glucose digestion. Based on the findings of McDougall et al. [62], it has been observed that the polyphenol extract derived from blueberries, containing a significant amount of anthocyanin, demonstrates inhibitory effects on the functions of α-amylase and α-glucosidase. The enhancement of glucose absorption by muscle and fat cells was observed when Zucker obese rats consumed freeze-dried blueberry powder. This was linked to the increased activity of the peroxisome proliferator-activated receptor-α (PPAR-α) and PPAR-γ in adipose tissue and skeletal muscle, as well as the improved transcription of PPARs contributed to glucose uptake/oxidation [33]. Refined anthocyanins have the potential to promote the secretion of insulin, leading to enhanced absorption of glucose in the muscles and fat cells [5]. Furthermore, the consumption of blueberry anthocyanins has been linked to improvements in insulin resistance [34]. Additionally, individuals with a diagnosis of type 2 diabetes often show elevated triglyceride levels in their bloodstream and heightened apolipoprotein C-III concentrations in their plasma. It has been proposed that focusing on apolipoprotein C-III may offer a new strategy for controlling diabetic dyslipidemia. The existence of anthocyanin has been demonstrated to have a notable effect on decreasing the levels of serum apolipoprotein B and C-III, leading to a reduction in serum triglyceride concentration [63]. Blueberry juice supplementation (25 g/kg) on high fat diet-induced prediabetes rat reduced fecal short-chain fatty acids (SCFAs), increased markers of arrested interscapular brown and epidydimal (iBAT) and energy expenditure, but aggravated lipotoxicity and hepatic steatosis and inhibited autophagy and endoplasmic reticulum (ER) stress responses in the liver [35]. Receiving an oral dose of 80 mg/kg of blueberry anthocyanins for approximately 12 weeks resulted in the alleviation of diabetes-related symptoms in STZ-induced diabetic rats, achieved through the activation of the nuclear factor E2-related factor 2 (Nrf2)/HO-1 signaling pathway [22].

### 3.4. Anti-Obesity

With the rise in people’s quality of life, obesity has increasingly emerged as a significant global health issue, often leading to metabolic syndrome [64]. Obesity is linked to an imbalance in energy, resulting in the excessive accumulation of fat tissue caused by a disparity between energy consumption and expenditure. Both conditions involve metabolic disturbances and are linked to metabolic syndrome [63]. Receiving blueberry extract has the potential to cause a notable decrease in the weight of diet-induced obese C57BL/6 mice. Blueberry anthocyanins not only have the ability to hinder the weight gain of mice, but also has a significant impact on reducing serum and liver lipids. Meanwhile, the hepatic SOD and GPx activity significantly increased but the expression of TNF-α, IL-6, and NF-κB was reduced in diet-induced obese mice [36]. The intake of lyophilized blueberry powder, juice, and purified anthocyanins at a 10% level resulted in a significant reduction in weight gain and body fat accumulation. It was discovered that the anti-obesity properties of blueberry anthocyanins were notably more effective than those of blueberry juice [37]. Interestingly, rats that were administered wild blueberry powder did not exhibit significant changes in weight when compared to the control group, but obese Zucker rats that were given wild blueberry powder demonstrated enhancements in specific aspects of glucose metabolism [30]. Another study found that mice that were administered freeze-dried blueberry powder showed an increase in body weight compared to the control group. Nevertheless, there was a notable enhancement in glucose intolerance and hepatic steatosis [38]. The results were inconsistent in obese individuals, as there was no significant weight change observed in those who ingested 50 g/d of freeze-dried blueberry powder for a period of eight weeks [39]. Nevertheless, there were improvements in certain indicators of metabolic syndrome, which were concretely reflected in a decrease in systolic and diastolic blood pressures, a reduction in plasma oxidized LDL and serum MDA levels, and an enhancement of hydroxynonena. Overweight individuals who replaced 50 g of carbohydrates with a 50 g serving of blueberries saw significant reductions in body weight, fat accumulation, and the levels of LDL and total cholesterol [40]. Clinical trials have demonstrated that blueberries can effectively control lipid metabolism, leading to improvements in metabolic syndromes associated with obesity [41]. Blueberry anthocyanins have been shown to improve adipocyte function, suggesting a potential for inhibiting obesity due to its close relationship with abnormal adipocyte function [36,41].

### 3.5. Anti-CVD

In many developed nations, CVD has emerged as a leading contributor to mortality [65]. CVD encompasses a range of conditions impacting the cardiovascular system, including hyperlipidemia, blood viscosity, atherosclerosis, and hypertension. The occurrence and development of CVD are significantly influenced by one’s dietary habits. Consuming fruits and vegetables have been demonstrated to have a protective impact on CVD, with anthocyanins identified as the effective component [64]. The consumption of blueberry anthocyanins has been associated with improved cardiovascular health (Table 2). Based on epidemiological, clinical, and animal studies, it is indicated that the anthocyanins found in blueberries may offer defense against cardiovascular issues by influencing different aspects of the vascular system. The impacts consist of triggering signaling for endothelial nitric oxide synthase (eNOS), decreasing oxidative damage, enhancing the pathways related to inflammation, and improving dyslipidemia [66]. They can enhance endothelial function, reduce blood pressure, and improve lipid profiles by lowering low-density lipoprotein cholesterol (LDL-C) and increase high-density lipoprotein cholesterol (HDL-C). These effects contribute to a reduced risk of atherosclerosis and other CVD [65]. The mean size of aortic sinus lesions and descending aorta in ApoE^-/-^ mice decreased by 39% and 58%, respectively, after being treated with 1% lyophilized blueberry powder for 20 weeks, compared to the control group [17]. A double-blind study on individuals conducted that the high level of cholesterol is an early indicator of atherosclerosis [42]. They discovered that blueberry-derived delphinidin-3-*O*-β-glucoside and cyanidin-3-*O*-β-glucoside can effectively decrease markers of inflammatory response, such as plasma lL-1β, serum high-sensitivity C-reactive protein (hsCRP), and soluble vascular cell adhesion molecule-1 (sVCAM-1). Additionally, they found that these anthocyanins can lower levels of LDL-C and increase HDL-C. 

### 3.6. Anti-Cancer

An earlier study projected an increase in the number of cancer cases to around 22 million by the year 2030 [67]. As a result, the research focus has shifted towards finding effective methods for cancer prevention. Blueberry has been extensively researched and demonstrated to possess anti-cancer properties (Table 2). According to Zhou et al. [43], the anthocyanin found in blueberries has been shown to greatly promote apoptosis in HepG-2 cells. Anthocyanin triggered programmed cell death in HepG-2 cells, leading to an elevation of the production of ROS, caspase-3 activity, and Bax expression, but a decrease in the mitochondrial membrane potential and Bcl-2 expression [43]. Certain chemical compounds found in blueberries such as anthocyanins have been recognized for their ability to combat cancer. Blueberry anthocyanins and polyphenol extracts have been found to boost the immune system and inhibit the proliferation of cancer cells [44]. Research has demonstrated that the growth of MDA-MB-231 and MCF7 breast cancer cells was effectively suppressed after being treated with 250 μg/mL blueberry anthocyanin extract and anthocyanin pyruvate adduct for 24 h [45]. Blueberry extract has been shown to effectively block cell growth and trigger programmed cell death via the COX-2 pathway in HT-29 colon cancer cells [46]. In a study using animal models, the extract of blueberry polyphenols was found to enhance certain immune functions and suppress the growth of tumors in mice with CD-1 tumors [47]. Jeyabalan et al. [48] discovered that giving ACI rats a 5% freeze-dried blueberry powder diet containing 21 mg/g of anthocyanin delayed the development of breast tumors and reduced both the number and size of tumors. European blueberry anthocyanins, including its individuals, were found to effectively suppress the growth of human lung cancer A549 and H1299 cells at concentrations ranging from 2.5 to 7.5% [50]. After a six-week daily consumption of blueberry fruit, 25 participants showed a notable increase in natural killer (NK) cells count (a specific kind of lymphocyte that focuses on irregular cellular immune reactions) and elevated levels of anti-inflammatory cytokine IL-10 [51]. The presence of 50 μM of blueberry—derived malvidin-3-glucoside and malvidin-3-galactoside—has the potential to decrease the inflammatory markers monocyte chemotactic protein-1 (MCP-1), intercellular adhesion molecule-1 (ICAM-1), and vascular cell adhesion molecule-1 (VCAM-1) production both in the protein and mRNA levels that are triggered by TNF-α. The process works by breaking down IκBα and moving p65 into the nucleus; thus, the NF-κB pathway is regulated, leading to its anti-inflammatory properties [52].

### 3.7. Neuroprotective

The prevalence and mortality rates of major depressive disorder make it one of the most common neuropsychiatric disorders. The pathogenesis of significant depression condition is intricate, and there are limited rehabilitation alternatives accessible. Blueberries contain a high number of polyphenols and have the potential to protect the nervous system [68]. Numerous studies suggest that blueberries and their anthocyanins act as natural agents for protecting nerve cells and reducing damage effectively. Tan et al. [53] discovered that the addition of extracts (200 mg/kg·bw/day) and cyanidin-3-*O*-galactoside (50 mg/kg·bw/day) from blueberries can enhance the memory for spatial information and controls the expression of hippocampal ERK in senescence-accelerated mice. A possible key component for these biological effects could be cyanidin-3-*O*-galactoside. During an experiment involving rats, researchers discovered that the ingestion of blueberry ethanol extract provided protection against the neurological and behavioral dysfunction caused by ketamine-induced mania, with physiological indicators including the enhancement of thiobarbituric acid reactive species formation (TBARS) and IL-6 level and the decrease in the total sulfhydryl (SH) content and activities of SOD, CAT, and GPx [54]. This finding supports the hypothesis that blueberries may have a protective effect against oxidative injury and behavioral impairments linked to bipolar disorder during periods of mania. Receiving blueberry supplementation prevented the alterations in oxidative stress markers caused by ketamine. The polyphenols found in blueberries can help prevent alcoholic fatty liver disease in C57BL/6 mice by enhancing the process of autophagy, which speeds up the breakdown of fats and removes excessive TG from liver cells [55]. Furthermore, a research study on nine older adults who drank wild blueberry juice for a period of 12 weeks demonstrated enhancements in their neurocognitive functions [56]. Another study found that the supplementation (~148 g/d) of blueberry, containing 14.53 ± 0.04 mg cyanidin 3-glucoside equivalents/g dry weight, can affect the blood oxygen level-dependent (BOLD) signal in older adults with mild cognitive impairment over a period of 16 weeks. This resulted in activation of the pre-central gyrus, middle frontal gyrus, and inferior parietal lobe on the left (corrected *p*  <  0.01), but there was no clear evidence of improvement in working memory associated with blueberry supplementation [57].

### 3.8. Vision Protection

Multiple studies have presented evidence suggesting that consuming blueberry anthocyanin may lead to an improvement in vision. The positive effects of blueberry anthocyanins on eyesight include improvements in diabetic retinopathy, age-related macular degeneration, night vision, and other advantages. The extract of blueberry anthocyanins, along with malvidin, malvidin-3-glucoside, and malvidin-3-galactoside, demonstrated the ability to reduce oxidative damage induced by H_2_O_2_ in human retinal pigment epithelial cells [18]. Song et al. [22] found that blueberry anthocyanins have a protective effect on retinal cells against oxidative injury and inflammation in diabetic rats, potentially through the regulation of the Nrf2/HO-1 signaling pathway. Wang et al. [58] found that anthocyanins (pelargonidin-3-glucoside, cyanidin-3-glucoside, delphinidin-3-glucoside, and malvidin-3-glucoside) in berries (blueberry, blackberry, and strawberry) have been shown to provide protection against damage caused by visible light in human RPE cells due to their diverse structures. One of the compounds, cyanidin-3-glucoside, structurally containing an ortho-hydroxyl group in its B-ring, has various protective effects (such as antioxidant, anti-angiogenic, and anti-aging) on RPE cells. Therefore, it could potentially be used as a preventive health supplement for retinal diseases. A study discusses two trials involving humans, which examine the influence of blueberry anthocyanins on the recovery of vision in low light conditions, night vision functionality, and adaptation to darkness following retinal photobleaching [59]. Trial-1 consisted of two doses of blueberry products (271 and 7.11 mg cyanidin 3-glucoside equivalents) employed under a shorter period (3-week treatment and 3-week washout) (n = 72). Trial-2 consisted of a dose equivalent of 346 mg cyanidin 3-glucoside of blueberry product employed under longer periods (8-week treatment and 4-week washout; 12-week treatment and 8-week washout) (n = 59). Neither adaptation to darkness nor night vision functionality showed improvement with blueberry anthocyanin intake in either Trial-1 or Trial-2. However, there was an obvious enhancement in the recovery of vision after photobleaching under the experimental doses (both *p* = 0.014, in Trial-1) and periods (*p* = 0.027, over 8 weeks intervention; *p* = 0.030, over 12 weeks intervention, in Trial-2). While anthocyanins did speed up the recovery of photobleaching, it remains unclear whether this enhancement would have any effect on daily visual function [59].

### 3.9. Others

There are also some reports on the effects of blueberry anthocyanins on microbial growth. It was discovered that consuming beverages containing blueberry powder for a period of eight weeks led to a rise in the percentage of Bifidobacteria within the gut microbiota of individuals [69]. Zimmer et al. [60] found that six hydroethanolic extracts of blueberry (*Vaccinium virgatum*), with a total anthocyanins content ranging from 40.98 to 62.92 mg cyanindin-3-glucoside/100 g fresh fruit, could against *Staphylococcus epidermidis* biofilm, showing up to 84% inhibition without impacting bacterial growth, and there was a direct relationship between the total phenolic content. Another study reported that a blueberry extract high in anthocyanins (184.7 μg/mg) could inhibit biofilm formation and bacterial adhesion for several pathogens (including *Acinetobacter baumannii*, *Escherichia coli*, *P. aeruginosa*, *Proteus mirabilis*, and *Staphylococcus aureus*) [61].

## 4. Stability of Blueberry Anthocyanins

**Figure 2:**
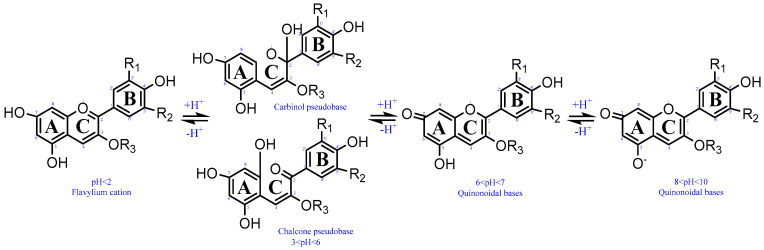
Effect of different pH levels on the structure of anthocyanins.

Environmental factors have the greatest impact on anthocyanins among the phenolics found in blueberries [9]. Anthocyanins are extremely labile, prone to degradation, and influenced by a multitude of factors such as their chemical composition, concentration, solvent environment, pH level, temperature fluctuations, ionic strength variations, exposure to light and oxygen, enzymatic activity, protein interactions, and the presence of metal ions. These factors can ultimately result in diminished color intensity, reduced biological efficacy, and decreased bioavailability [70,71,72]. The ionic properties of anthocyanins enable their molecular configuration to be altered in response to the surrounding pH levels, leading to a diverse range of colors and shades (Figure 2) [73]. The stability of both anthocyanins was observed to decrease gradually as the pH increased [71]. Their stability is observed within the pH range of 2–3, but becomes unstable within the pH range of 6–8, resulting in a reduction in their availability for biological processes. It was suggested that the pH sensitivity of anthocyanins is due to the ease with which the pyryum ring of the anthocyanin skeleton opens up and forms a chalcone structure, leading to eventual degradation of the anthocyanin. The heat resistance of anthocyanins in blueberries diminishes as temperature rises during processing and storage. The process of heat treatment yields a rich brown product, which suggests degradation, especially when oxygen is present [72]. In certain instances, glycosides can be acylated by acetic acid, ferulic acid, caffeic acid, *p*-coumaric acid, or synephrine. The existence of this presence allows the anthocyanin molecule to remain stable even in highly acidic or hot environments. The stability of cyanidin in acetonitrile was found to be highly unstable, with a slow initial degradation rate, but nearly 60% degraded within 5 h. In contrast, methanol showed better stability, with only a slight decline of cyanidin and 80% of the compound remaining intact. Interestingly, complete stability was achieved when using acidified methanol, as no degradation or loss was observed even after 5 h at room temperature [74].

## 5. Bioavailability of Blueberry Anthocyanins

The United States Food and Drug Administration (FDA) defines bioavailability as the measure of how quickly and thoroughly the active component or element is taken up into the bloodstream and reaches the site where it exerts its effects [3]. The absorption of anthocyanins relies on factors such as their chemical structure, the amount consumed, and the length of exposure. Any unabsorbed compounds are moved to the colon where they can undergo further changes or be utilized as prebiotics. It was also documented that microbiota may metabolize anthocyanins and that they or their byproducts can influence the proliferation of specific bacteria [75]. However, the lack of stability in anthocyanins results in limited bioavailability, and their breakdown in water-based solutions leads to both color loss and a decrease in biological activity [70,71]. Certain anthocyanins capable of withstanding gastrointestinal pH fluctuations can be absorbed in their entirety. However, their absorption is influenced by the wide array of molecular sizes, the presence of glycosides, the length of sugar moieties, and the inclusion of acyl groups [76].

Although anthocyanins are generally considered to be less bioaccessible, blueberries may offer a higher level of absorbable anthocyanins compared to fruits like raspberries and blackberries [77,78]. The different bioaccessibility of anthocyanins in different fruits may be related to specific protective properties or unique food matrices, such as fibers that are either soluble or insoluble and have the ability to interact with anthocyanins. Table 3 lists the data concerning the bioavailability of blueberries and their anthocyanins. It was reported that during the simulated intestinal digestion of Caco-2 monolayers, approximately 42% of total anthocyanins from Chinese wild blueberries were lost (35.60% of cyanidin-3-galactoside, and 57.64% of delphinidin-3-glucoside) [76]. Structurally, a reduction in the number of hydroxyl groups on the B-ring and the addition of methoxy groups to the glycosidic hydroxyl group resulted in enhanced stability of anthocyanins. Additionally, the increased hydrophobicity of the anthocyanins led to improved uptake into cell monolayers.

In vivo data showed that delphinidin and its acid-hydrolyzed glycosidic elements were detected in lung tissue of female thymus-less mice after they were placed on a diet containing 5% (*w*/*w*) of blueberry powder for 10 days of treatment, suggesting that anthocyanins can reach tissues outside the gastrointestinal tract and exert their effects [74]. Anthocyanins and their breakdown products were found in the urine of ovariectomized Sprague Daley rats that were given a diet with 5% (*w*/*w*) blueberries. Cyanidins and malvidins remained mostly unchanged, while delphinidins and peonidins underwent significant metabolism to form glucuronide and sulphated forms, a minimal quantity of all six anthocyanins was identifiable in the plasma [79]. Another human experiment found that the relative bioavailability of ^13^C_5_-labeled cyanidin-3-glucoside (500 mg/day) was 12.38 ± 1.38% (with 5.37 ± 0.67% excreted in urine and 6.91 ± 1.59% exhaled in breath). Its metabolites including phenolics, phenylacetic, hippuric, and phenylpropionic acid in blood, urine, breath, and feces peaked at 30 min, 0~1 h, 6 h, and 6~24 h after ingestion, respectively [80]. Several human trial studies have shown that anthocyanins derived from whole blueberries or blueberry juice can be absorbed and detected in blood plasma and urine, indicating their bioavailability, indicating the mode of anthocyanin dissemination in the body [81,82,83]. One finding indicated that middle-aged men can readily absorb intact glycosylated and possibly acylated forms of anthocyanins shortly after consuming blueberries. Additionally, the existence of anthocyanins in the bloodstream might play a role in boosting ex vivo serum antioxidant levels induced by dietary factors. It has been noted that the absorption of blueberry anthocyanins does not only involve the compounds themselves, but also their metabolized products, which are likely to exhibit biological activity.

## 6. Applications of Blueberry Anthocyanins

### 6.1. Color Indicator

Anthocyanin, a vibrant natural food dye, is gaining attention in packaging technology for its ability to change color based on environmental pH levels. Recent studies have highlighted biopolymer-based smart packaging films enhanced with anthocyanins (Table 4) [84]. These pH-responsive films allow for real-time visual monitoring of product quality. The color-changing properties of anthocyanins are extensively utilized in creating color indicator films for various food packaging applications [85]. These films are advantageous for on-the-spot evaluation of food safety and quality. Additionally, anthocyanin-enhanced packaging films possess strong antioxidant properties that aid in the prevention of color alteration and lipid oxidation in packaged food items. There is great potential for intelligent pH-responsive packaging films to play a promising role in maintaining product quality and safety in the food industry. These innovative films offer new opportunities for both producers and consumers. One of the primary uses of blueberry anthocyanins is as a natural color indicator [86]. Because of their vibrant hues and safety profile, they are used to make various biopolymer-based smart packaging films for food (such as shrimp and milk) freshness monitoring [87,88,89,90].

### 6.2. Functional Foods and Supplements

Functional foods and beverages are formulated to provide health benefits beyond just basic nutrition by including blueberry anthocyanins (Table 4) [23,69]. These products capitalize on the health-promoting properties of anthocyanins, appealing to health-conscious consumers. One study suggests that incorporating whole blueberries into the diet may be an effective approach for enhancing glucose regulation in inactive young people [29]. Both the immediate consumption of blueberries and brief supplementation showed beneficial impacts on managing glucose levels and insulin levels in sedentary individuals. Glucose concentrations were reduced by acute intake, and there was a trend towards lower insulin levels with short-term supplementation.

## 7. Conclusions

At present, 25 kinds of anthocyanins have been identified in blueberry. Blueberry anthocyanins offer a range of functional capabilities that are beneficial for health, including antioxidant, anti-inflammatory, anti-CVD, neuroprotective, and anti-diabetic effects. Blueberry anthocyanins’ stability is impacted by many factors such as pH, temperature, and exposure to light, resulting in their unstable availability for biological processes. The bioavailability of blueberry anthocyanins varies in different individuals and organs, and is mainly partially utilized in the intestinal segment. They are used as natural color indicator and functional ingredients. However, future research should focus on improving stability and exploring innovative applications to maximize the functional properties of blueberry anthocyanins.

## Figures and Tables

**Table 2:** Health benefits of blueberry anthocyanins.

Benefits	Treatment	Evaluation Items	Reference
Antioxidant	High-purity blueberry anthocyanins (containing 14 anthocyanins)	EC_50_ value of ABTS^•+^ radical scavenging = 14.99 μg/mL; EC_50_ value of DPPH radical scavenging = 26.48 μg/mL	[15]
	Extracts of blueberry anthocyanin form ten varieties (5, 10, 20 μg/mL) against acrylamide-induced toxicity in HepG2 cells	blueberry anthocyanin extracts from Polaris: ↑ cell viability, ↑ T-SOD, ↑ CAT, ↓ MDA	[16]
	ApoE^−/−^ mice were given a diet of AIN-93G containing 1% freeze-dried whole blueberries for a period of 20 weeks	↑ SOD 1, ↑ SOD2, ↑ GSR, ↑ TXNRD1 in aorta	[17]
	Extracts of blueberry anthocyanin, especially malvidin, malvidin-3-glucoside, and malvidin-3-galactoside (all at 5 μg/mL) against H_2_O_2_-induced oxidative damage in human RPE cells	↓ MDA, ↓ ROS ↑ SOD, ↑ CAT, ↑ GPx, ↑ cell viability, ↓ MAPK pathways (ERK1/2 and p38), ↓ VEGF, ↑ Akt signal pathways	[18]
	Delphinidin (50 μM) on high glucose-induced mesangial cells	↓ cell proliferation, ↓ NADPH oxidase 1 (NOX-1), ↓ mitochondrial superoxide	[19]
	Malvidin, malvidin-3-galactoside and malvidin-3-glucoside or mixture of the two glycosides (1, 5, and 10 µmol/L)	↓ ROS, ↓ XO-1, ↑ SOD, ↑ HO-1	[20]
	Blueberry anthocyanin (80, 40, 20, 10, 5, and 2.5 μg/mL) on human umbilical vein endothelial cells (HUVECs)	↑ cell viability and ↓ apoptosis rate through Bax- and Caspase 3-dependent pathways	[21]
	Blueberry anthocyanins (20, 40, and 80 mg/kg) were given orally on streptozotocin (STZ, 60 mg/kg)-induced diabetes rat for ~12 weeks	↑ GSH, ↑ GPx, ↓ MDA, ↓ ROS	[22]
	Blueberry anthocyanins extract (50, 150, 250 mg/kg) was given by oral administration once daily to acrylamide-induced diverse toxicity mice for 14 days	↓ ROS, ↑ GSH, ↓ GPx, ↑ GST, ↑ GPx1, ↑ GST1, ↑ γ-GCS, ↓ CYP2E1	[23]
Anti-inflammatory	The polyphenol-rich, anthocyanin-rich, and proanthocyanidin-rich extract of blueberry on lipopolysaccharide-induced murine RAW 264.7 macrophages	↓ COX-2, ↓ iNOS, and ↓ IL-1β	[24]
	Blueberry extract with an anthocyanin content of 0.58 mg/mL was given to collagen-induced arthritis rats	↓ bone absorption, ↓ osteophyte formation, and ↓ soft tissue swelling	[25]
	Blueberry-derived cyanidin-3-glucoside and pectin complexes were administered to mice with DSS-induced colitis	↓ pro-inflammatory factors, ↑ anti-inflammatory factor levels, ↓ NF-κB signaling pathway, ↑ Bcl-2/Bax and caspase-3/cleaved caspase-3 genes, ↑ gut microbiota composition	[26]
	Total flavonoids and their classes (anthocyanins, flavonols, flavanones, flavan-3-ols, polymers, and flavones) were assessed through a cross-sectional analysis of 2375 Framingham Heart Study Offspring Cohort participants	↓ inflammation score (subgroups of functionally related biomarker scores including acute phase reactants, cytokines, and oxidative stress)	[27]
	Blueberry anthocyanins (80 mg/kg) were given orally on STZ-induced diabetes rat	↓ VEGF, ↓ IL-1β	[22]
	The polyphenol-enriched extracts (from the sera of rats after fed with AIN-93G diet containing 10% freeze-dried blueberries) were treated on RAW264.7 macrophages	↓ IL-6, ↓ TNF-α, ↓ IκB and NF-κB p65, ↓ MAPK p38 and JNK	[28]
Anti-diabetes	The subject ingested 150 g of blueberries and 150 g of white bread, followed by blood sample collection at 0, 30, 60, 90, and 120 min for the measurement of glucose, insulin, and plasma antioxidant capacity	↓ insulin levels, ↑ plasma GSH	[29]
	8% enriched wild blueberry consumption in the obese Zucker rat for 8 weeks	↓ HbA1c, ↓ RBP4, ↓ resistin	[30]
	Administration of a phenolic-rich extract (containing 287.0 ± 9.7 mg/g anthocyanins) and an anthocyanin-enriched fraction (containing 595 ± 20.0 mg/g cyanidin-3-glucoside equivalents) at a dose of 500 mg/kg, as well as administration of pure anthocyanins delphinidin-3-*O*-glucoside and malvidin-3-*O*-glucoside at a dose of 300 mg/kg, were performed in diabetic C57b1/6J mice	↓ blood glucose levels by 33% and 51%	[31]
	Blueberry consumption (22 g/d) in men with type 2 diabetes	↓ hemoglobin A1c, ↓ fructosamine, ↓ triglycerides, ↓ aspartate transaminase, ↓ alanine transaminase	[32]
	A higher-fat diet (45% of kcal) or a lower-fat diet (10% of kcal) containing 2% (wt/wt) freeze-dried whole highbush blueberry powder were given to Zucker Fatty and Zucker Lean rats	↓ triglycerides, ↓ fasting insulin, ↓ glucose, ↓ abdominal fat mass, ↑ PPARs, ↓ liver and body weight, ↓ total fat mass	[33]
	Blueberry anthocyanins (160 mg) were given to 58 diabetic patients twice daily for 24 weeks	↓ LDL-C, ↓ triglycerides, ↓ apolipoprotein (apo) B-48, ↓ apo C-III, ↑ HDL-C, ↓ 8-iso-prostaglandin F2α, ↓ 13-hydroxyoctadecadienoic acid, and ↓ carbonylated proteins	[34]
	Blueberry juice supplementation (25 g/kg) on high fat diet-induced prediabetes rat	↓ fecal SCFAs, ↑ iBAT thermogenesis and energy expenditure, ↑ lipotoxicity and hepatic steatosis, ↓ autophagy and ER stress responses	[35]
	Blueberry anthocyanins (20, 40, and 80 mg/kg) were given orally on STZ-induced diabetes rat	↑ Nrf2/HO-1 signaling	[22]
Anti-obesity	Blueberry anthocyanin supplementation (200 mg/kg) on a high-fat-diet-induced obese C57BL/6 mice for 12 weeks	↓ weight gain, ↓ serum and liver lipids, ↑ SOD and GPx, ↓ TNF-α, IL-6, and NF-κB	[36]
	The administration of freeze-dried blueberry powder, juice, and purified anthocyanins at a concentration of 10% to C57BL/6J mice with high-fat diet-induced obesity for a duration of 8 weeks	↓ weight gain, ↓ body fat, the anti-obesity potential of blueberry anthocyanins was superior to that of blueberry juice	[37]
	Obese Zucker rat given 8% lyophilized blueberry powder (total anthocyanins content was 1.5% *w*/*w*, most abundant in malvidin 3-galactoside and peonidin 3-glucoside) for 8 weeks	↓ resistin expression, ↓ RBP4 expression	[30]
	Menopausal mice were given a high-fat diet or supplemented with 4% blueberry powder for 12 weeks	↓ glucose intolerance and hepatic steatosis in obese postmenopausal mice, regardless of the individual‘s weight	[38]
	50 g of lyophilized blueberry powder daily in obese individuals [4 males and 44 females; BMI at 37.8 ± 2.3 kg/m^2^; age at 50.0] for 8 weeks	↓ systolic and diastolic blood pressures, ↓ plasma oxidized LDL and serum MDA, ↑ hydroxynonena	[39]
	Blueberry fruit was given to obese patients over a 12 week period of time	↓ body weight (11–14% more in males), ↓ body fat (3–1.4% more in females), ↓ LDL and total cholesterol	[40]
	The primary 10 types of anthocyanins from blueberry against free fatty acid (FFA)-induced NAFLD in L02 cells	↓ lipid accumulation through the Nrf2/ARE signaling pathway	[41]
Anti-CVD	ApoE ^−/−^ mice were given a diet of AIN-93G containing 1% freeze-dried whole blueberries for a period of 20 weeks	↓ average area of aortic sinus lesions and descending aorta (39% and 58%, respectively)	[17]
	A group of 150 individuals diagnosed with high cholesterol were given either a purified anthocyanin mixture (320 mg/d) or a placebo twice daily for a period of 24 weeks	The purified anthocyanins delphinidin-3-*O*-β-glucoside and cyanidin-3-*O*-β-glucoside from blueberries: ↓ serum hsCRP, sVCAM-1, and plasma lL-1β, ↓ LDL-C, ↑ HDL-C	[42]
Anti-cancer	Blueberry anthocyanins induced cell apoptosis in human hepatocellular carcinoma HepG-2 cells	↑ ROS, ↑ caspase-3, ↓ Bcl-2, ↑ Bax ↑ p38 MAPK and p53, ↓ TGF-β	[43]
	Blueberry anthocyanin crude/purified extract and blueberry polyphenols crude/purified extract were, respectively, investigated in cyclophosphamide-induced female Balb/c mice and in human breast cancer cell	Crude extract > pure extract, anthocyanin + polyphenol crude product mixture showed a more powerful tumor suppressor, also more efficient at improving immune function	[44]
	Blueberry anthocyanin extract (250 μg/mL) and anthocyanin pyruvate adduct were treated in two breast cancer cell lines (MDA-MB-231 and MCF7) for 24 h	↓ cell proliferation (both)	[45]
	Extracts (25 to 200 μg/mL) of six popularly consumed berries (blackberry, black raspberry, blueberry, cranberry, red raspberry, and strawberry) were evaluated in human oral (KB, CAL-27), breast (MCF-7), colon (HT-29, HCT116), and prostate (LNCaP) tumor cell lines	↓ cell growth and ↑ cell death via the COX-2 pathway, IC_50_ values of blueberry extracts on CAL-27, HT29, MCF-7, KB, HCT116, LNCaP = 177.40, 89.96, 169.90, 171.30, 90.00, 36.45, respectively	[46]
	Crude and purified blueberries polyphenol extracts tested on CD-1 tumor-bearing mice	↓ growth of tumors and ↑ immunity	[47]
	Administering a 5% freeze-dried blue-berry powder diet with an anthocyanin content of 21 mg/g to 17β-estradiol (E_2_)-mediated mammary tumorigenesis ACI rats either 2 weeks prior to or 12 weeks after E_2_ treatment in preventive and therapeutic groups, respectively	↓ onset of breast tumors and ↓ variety and size of tumors, ↓ CYP 1A1 and ER-α ↓ microRNA (miR-18a and miR-34c)	[48]
	A blueberry preparation with high levels of polyphenols and a non-fermented version were tested in three different types of cells (murine 4T1 and human MCF7 and MDA-MB-231) and in BALB/c mice	↓ tumor development, ↓ mammospheres, and ↓ lung metastasis by PI3K/Akt, MAPK/ERK and STAT3 pathways	[49]
	European blueberry anthocyanins (supplemented with 2.5–7.5%) were tested on human lung cancer cells (A549 and H1299)	Inhibition: ① The mixture of blueberry anthocyanins (5%, *w*/*w*) and black raspberry anthocyanins (2.5%) > blueberry anthocyanins alone (71% vs. 42%) ② A combination of blueberry delphinidin and black raspberry punicalagins > delphinidin	[50]
	25 participants were instructed to consume 250 g of blueberries daily for 6 weeks, as well as an additional 375 g one hour before engaging in a 2.5 h running session	↑ NK cells and IL-10	[51]
	Blueberry-derived malvidin-3-glucoside and malvidin-3-galactoside were tested on HUVECs	↓ MCP-1, ICAM-1 and VCAM-1 ↓ IκBα and ↑ p65 (↓ NF-κB pathway)	[52]
Neuroprotective	Blueberry extracts (200 mg/kg·bw/day) and cyaniding-3-*O*-galactoside (50 mg/kg·bw/day) from blueberry were given to senescence-accelerated mice prone 8 (SAMP8) mice for 8 weeks	↓ cellular injury, ↑ hippocampal neurons survival, ↓ pyramidal cell layer damage, ↑ SOD, ↓ MDA, ↑ p-ERK	[53]
	Blueberry extract (200 mg/kg, once a day for 14 days) were examined in ketamine-induced hyperactivity rats	↓ TBARS, ↑ Total SH, ↑ SOD, ↑ CAT, ↑ GPx	[54]
	Blueberry polyphenols were examined in C57BL/6 mice with alcoholic fatty liver disease	↑ hepatocytes autophagy at 200 mg/kg concentration	[55]
	A group of nine elderly individuals experiencing initial memory decline were given wild blueberry juice on a daily basis over the course of 12 weeks	↑ paired associate learning (*p* = 0.009) and word list recall (*p* = 0.04), ↓ depressive symptoms (*p* = 0.08) and glucose levels (*p* = 0.10)	[56]
	Daily supplementation (approximately 148 g) of blueberry (with an anthocyanin content of 14.53 ± 0.04 mg cyanidin 3-glucoside equivalents/g dry weight) on blood oxygen level-dependent (BOLD) signal in older adults with mild cognitive impairment for 16 weeks	↑ left pre-central gyrus, left middle frontal gyrus, and left inferior parietal lobe (corrected *p* < 0.01), however, there is not a definitive sign of improved working memory linked to the addition of blueberries	[57]
Vision protection	Extracts of blueberry anthocyanin, especially malvidin, malvidin-3-glucoside, and malvidin-3-galactoside (all at 5 μg/mL) were tested in human RPE cells	↓ MDA, ↓ ROS ↑ SOD, ↑ CAT, ↑ GPx, ↑ cell viability, ↓ MAPK pathways (ERK1/2 and p38), ↓ VEGF, ↑ Akt signal pathways	[18]
	Blueberry anthocyanins (20, 40, and 80 mg/kg) were given orally on STZ-induced diabetes rat for ~12 weeks	↓ retinal abnormalities and ↓ development of diabetic retinopathy	[22]
	Blueberry-derived pelargonidin-3-glucoside, cyanidin-3-glucoside, delphinidin-3-glucoside, and malvidin-3-glucoside against visible light-induced damage in human retinal pigment epithelial cells	↓ ROS, ↓ VEGF, ↓ β-galactosidase (antioxidant, anti-angiogenic and anti-aging)	[58]
	Trial-1: Two dose of blueberry products (271 and 7.11 mg cyanidin 3-glucoside equivalents) employed under a shorter period (3-week treatment and 3-week washout) (n = 72); Trial-2: A dose equivalent of 346 mg cyanidin 3-glucoside of blueberry product employed under the longer periods (8-week treatment and 4-week washout; 12-week treatment and 8-week washout) (n = 59).	① Neither dark adaptation nor night vision was improved in two trials. ② ↑ vision recovery at two doses (*p* = 0.014) in Trial-1, and after 8 weeks (*p* = 0.027) and 12 weeks (*p* = 0.030) treatments in Trial-2.	[59]
Others	In vitro anaerobic fermentation of high-purity blueberry anthocyanins (containing 14 anthocyanins) by human fecal micro-organisms	Increase the relative abundances of some certain communities including *Bifidobacterium* spp.	[15]
	Six hydroethanolic extracts of blueberry rabbit-eye (*Vaccinium virgatum*), with a total anthocyanins content ranged from 40.98 to 62.92 mg cyanindin-3-glucoside/100 g fresh fruit) against *Staphylococcus epidermidis* and *Pseudomonas aeruginosa*	Inhibiting up to 84% of *S. Epidermidis* biofilm formation without affecting bacterial growth, while showing a linear relationship with the total phenolic content	[60]
	A blueberry extract with a high concentration of anthocyanins (184.7 µg/mg extract) was tested for its effects on the growth, adhesion, and biofilm formation of various pathogens (*Acinetobacter baumannii*, *Escherichia coli*, *P. aeruginosa*, *Proteus mirabilis*, *and Staphylococcus aureus*)	Inhibiting biofilm formation and bacterial adhesion for all micro-organisms tested hindering the growth of *S. aureus* and *E. coli*	[61]

↑ indicates the promotion trend, and ↓ indicates the downward trend.

**Table 3:** Evaluation of bioavailability of blueberries and their anthocyanins.

Evaluation Model	Treatment	Bioavailability	Reference
In vitro Caco-2 monolayers	Chinese wild blueberries	Approximately 42% of the total anthocyanin lost (35.60% in cyanidin-3-galactoside, 57.64% in delphinidin-3-glucoside)	[76]
Female athymic nude mice	5% blueberry powder diet (*w*/*w*), 10 mg/mouse; po	Anthocyanins can be detected in lung tissue and are bioavailable beyond the gastrointestinal tract	[74]
Ovariectomized Sprague-Daley rats	Diet containing 5% (*w*/*w*) blueberries, 0–1000 mg/kg, po	Smaller amounts of anthocyanins are detected in urine within 24 h and dose-dependent	[79]
Healthy male participants	500 mg single oral bolus dose of ^13^C_5_-labeled cyanidin-3-glucoside	12.38 ± 1.38% (5.37 ± 0.67% excreted in urine and 6.91 ± 1.59% in breath)	[80]
Nine healthy participants	250 g of fresh blueberries either as the whole fruit or after juicing	A higher range of phenolic and other metabolites in plasma and urine 2 h after consumption of both whole and juiced blueberries	[81]
Human trial (ages 24–60 years, 13 women and 4 men)	250 mL blueberry juice containing 448 μmol cyanidin-3-glucoside equivalents	Cumulative excretion 79.3% of total anthocyanins (24–0 h)	[82]
Twelve participants (20–45 years of age and BMI of 25 to 33 kg m^−2^)	Wild blueberries beverage (25 g freeze dried powder)	Anthocyanins (1.1%) and cyanidin-3-glucoside (0.2%). Peaked ≈ 2 h totally post ingestion Peaked ≈ 2.6, 6.3, 7 and 8.8, respectively, for peonidins, delphinidins, cyanidins and petunidins	[83]

**Table 4:** Applications of blueberry anthocyanins.

Forms of Applications	Anthocyanin Objects	Evaluation Items	Reference
Indicator sensor for intelligent packaging	Cyanidin	The incorporation of 5, 10, and 15 wt% into blended films of quaternary ammonium chitosan/gelatin for monitoring the freshness of shrimp and milk	[89]
	Blueberry residue	Starch-based films developed by thermocompression	[88]
	Blueberry extract	Development of bionanocomposite films using corn starch and natural as well as modified nano-clays	[87]
	Blueberry residue	Replaced various simulants and foodstuffs with cassava starch film	[90]
A nutraceutical or a dietary supplement	Blueberry anthocyanins extract	↓ CYP2E1, ↑ GSH, ↓ GPx, ↑ GST, ↑ GPx1, ↑ GST1, ↑ γ-GCS, ↓ ROS	[23]
A dietary approach for enhancing glucose regulation in inactive young individuals	Whole blueberries	↓ insulin levels, ↑ plasma GSH	[29]
Wild blueberry powder drink	Wild blueberry drink	↑ *Bifidobacterium* spp. ↑ *Lactobacillus acidophilus*	[69]

↑ indicates the promotion trend, and ↓ indicates the downward trend.

## Data Availability

The original contributions presented in the study are included in the article, further inquiries can be directed to the corresponding author.
